# Down Syndrome in British Maternity Care: Mothers' Experiences of Prenatal Testing and Receiving a Prenatal or Postnatal Diagnosis

**DOI:** 10.1111/jar.70160

**Published:** 2025-12-08

**Authors:** Tamar Rutter, Richard Hastings, Nicola Enoch, Samantha Flynn, Matthew Randell, Chris Stinton

**Affiliations:** ^1^ Warwick Medical School University of Warwick Coventry UK; ^2^ Intellectual Disabilities Research Institute University of Birmingham Birmingham UK; ^3^ Down Syndrome UK Leamington Spa UK; ^4^ Centre for Research in Intellectual and Developmental Disabilities University of Warwick Coventry UK

**Keywords:** diagnosis, down syndrome, maternity care, parental experiences, prenatal screening, prenatal testing

## Abstract

**Background:**

Parental experiences of prenatal testing and receiving a Down syndrome diagnosis for their child are impacted by the quality of information and support available. We explored the care experiences of mothers living in Great Britain who received a prenatal or postnatal Down syndrome diagnosis.

**Method:**

Mothers (*N* = 280) of children with Down syndrome born between 2019 and 2022 completed an online survey about being offered prenatal diagnostic testing and receiving a prenatal or postnatal diagnosis.

**Results:**

Qualitative findings underscored the impact of sensitive, supportive care and communication on participants' sense of autonomy and emotional adjustment. However, many participants felt information about Down syndrome was imbalanced, outdated, or insufficient. Some also reported feeling pressured to undergo diagnostic testing or pregnancy termination.

**Conclusions:**

The findings highlight the importance of communication that prioritises individual needs, and of ensuring healthcare professionals are equipped to support parents as they begin their parenting journey.

## Summary

- When parents are told that their child has Down syndrome, good communication and support are essential to help them understand, adjust, and prepare.
- We found that some mothers felt pressured to have tests during pregnancy to confirm if their baby has Down syndrome. Feeling under pressure was upsetting and undermining.
- Mothers felt more confident when their decisions were supported by professionals and there was a welcoming attitude to their baby having Down syndrome.
- Most mothers were not given enough information to help them understand what the diagnosis meant. This could leave them feeling unprepared and alone. Healthcare professionals need up‐to‐date resources and training so they can support families at this critical time.

## 1 Introduction

Prenatal testing for Down syndrome is available as part of maternity healthcare provision in many countries (Sebire et al. 2024). In Great Britain, the prenatal diagnostic pathway begins with the offer of prenatal screening tests which identify those with a higher chance of having a baby with Down syndrome (Wilmot et al. 2023). In England, Scotland, and Wales, invasive diagnostic testing is available in the National Health Service (NHS) to women who receive higher chance results from initial screening tests (via serum markers and ultrasound examination) and/or from the ‘second line’ non‐invasive prenatal test (NIPT) (Sebire et al. 2024). In these countries, the law permits abortion on the grounds of ‘serious handicap’, which includes Down syndrome, at any gestational age (Sheldon 2024). NHS maternity care provision includes financial coverage for both prenatal testing and pregnancy termination. In Great Britain, the majority of pregnant women accept the offer of prenatal screening. National statistics for 2023–24 show that at least 88% of eligible women in England elected to pursue the initial screening test for Down syndrome and/or Edwards' and Patau's syndrome (NHS England 2025). In England, there were 700 live births of babies with Down syndrome in 2021, with 973 (87.3%) of pregnancies with a confirmed prenatal Down syndrome diagnosis ending in termination (NHS England 2024).

Prenatal testing is intended to enhance reproductive autonomy by allowing expectant parents to make informed decisions about their pregnancy (Ravitsky 2017). To achieve this, parents receiving a Down syndrome screening result or diagnosis need to understand what this means, and how it relates to their own lives and concerns. However, many parents who received prenatal and postnatal diagnoses have reported receiving insufficient information about Down syndrome at this crucial time (Crombag et al. 2020; Goodwin et al. 2015; Smith et al. 2019). Decisions about prenatal diagnosis and pregnancy continuation can be highly stressful and complex, with emotional aspects such as shock and anxiety adding to the difficulty of assimilating and considering necessary information (Durand et al. 2010; St‐Jacques et al. 2008). Time is an important resource for both emotional processing and conscious consideration (Sooben 2010), but previous research has found that some parents felt rushed into making decisions about prenatal testing and termination (Artal et al. 2025; Asgarova 2019). Following prenatal diagnosis, some parents have reported that healthcare professionals assumed they would terminate the pregnancy (Lou et al. 2020), or encouraged them to do so (Artal et al. 2025). Evidence shows that the way the diagnosis is communicated is typically highly memorable, with this experience carrying long‐term implications for parents' adjustment and mental wellbeing (Kratovil and Julion 2017; May et al. 2020; Mugweni et al. 2021).

There is a growing body of international research about parental experiences surrounding a diagnosis of Down syndrome. However, it is unclear to what extent the findings described above may generalise to recent experiences in the context of a national and publicly funded prenatal screening programme such as that available in Great Britain. Here, the evaluative introduction of NIPT to the NHS screening pathway reinvigorated attention to the social and ethical implications of prenatal testing for Down syndrome, raising public awareness and prompting updated training efforts for professionals (Nuffield Council on Bioethics 2016; Oxenford et al. 2017). Given the impact on practice of local professional guidelines and education, and nuances in the ethical principles emphasised in different national contexts (Benachi et al. 2020; Perrot and Horn 2022), it is important to understand recent parental experiences in specific settings such as this. Furthermore, many studies have provided crucial in‐depth insights by engaging with a small number of parents. As such, they have been unable to indicate a range or pattern of experiences across a wider group—though some notable exceptions are survey studies from the USA (e.g., Artal et al. 2025) and the Netherlands (Crombag et al. 2020). Therefore, by using survey methods to collect both qualitative and quantitative data, the primary aim of this study was to provide a comprehensive understanding of the range of experiences of mothers who received a prenatal or postnatal diagnosis of Down syndrome within NHS maternity care. By investigating their care journeys, including support in relation to both decision‐making and the diagnostic process, we sought to highlight key aspects of positive practice, and identify opportunities to improve early parental experiences.

## 2 Methods

### 2.1 Study Design

A cross‐sectional survey design was employed. Data were collected via a self‐completed online survey including closed and open‐ended questions. Open‐ended questions were used to provide additional insights into individual experiences and how they were understood by participants.

### 2.2 Participants

Participants were eligible to take part if they were the birth mother (18 years+) of a child with Down syndrome 2 months or older and born between 2019 and 2022. Participants were eligible if they were living in England, Scotland, or Wales during their pregnancy.

### 2.3 Survey

The questionnaire was adapted from a survey about the maternity experiences of mothers of children with Down syndrome previously conducted by Down Syndrome UK 2019. Three mothers of children with Down syndrome were consulted for their views on whether the adapted survey was coherent and relevant to address a sufficient range of significant experiences. Amendments were made based on their feedback. Three different mothers of children with Down syndrome then tested the survey and gave feedback about its length, clarity, sensitivity, and the available response options, with no further changes being indicated. Ethnicity, religion, and demographic questions were based on ONS guidelines (Office for National Statistics 2016), and questions about financial wellbeing were based on those used in a previous study (Hastings et al. 2020).

The survey (see Supporting Information S1), hosted on Qualtrics, addressed experiences of support, information, and communication from healthcare providers in relation to prenatal testing and prenatal or postnatal diagnosis, with additional questions about overall maternity care experiences in relation to Down syndrome. At the end of the survey, demographic questions were presented. Only the findings about diagnosis experiences are presented here; these pertain to the offer of prenatal diagnostic testing with amniocentesis or chorionic villus sampling, discussions about pregnancy termination, and receiving a prenatal or postnatal diagnosis. Findings about experiences of prenatal screening, which include initial screening tests and NIPT, are reported in Rutter et al. (2025).

Most survey questions were multiple‐choice. Open‐ended questions were presented at the end of each section for participants to optionally elaborate on their responses. Participants took differing pathways through the survey, depending upon their answers to questions about their prenatal testing experiences and choices. Answering each survey question was optional.

### 2.4 Procedure

Recruitment (August 2022–February 2023) was conducted by two UK charitable organisations (Down Syndrome UK and Cerebra) who promoted the study via their closed parent networks on social media and their email lists/newsletters. The survey was not indexed on search engines. There was no remuneration or other financial incentive available for participating. Participants were first presented with study information, followed by consent and eligibility questions, and then the main survey questions. Given the distribution method, the overall response rate is unknown.

### 2.5 Analysis

Quantitative data analysis used IBM SPSS Statistics (Version 28) and was primarily descriptive. Analysis was conducted on an item‐by‐item basis; data from all respondents who completed the relevant question(s) were included in each analysis.

Qualitative data were analysed using Lumivero NVivo (Release 1.0), following the framework method (Ritchie and Spencer 2002). The research questions provided initial thematic domains (support with decisions, and receiving news about Down syndrome), with an initial coding framework developed through data familiarisation and in line with the survey questions. This was completed by the first author in consultation with a co‐author with qualitative expertise. Two researchers began applying the framework to code the data in parallel, meeting frequently to compare and discuss coding and agree upon changes to the framework. After coding 10% of the data this way and agreeing that an adequate level of consistency had been achieved, one researcher continued to ‘index’ the remaining data using the agreed framework. Matrices were then produced wherein the data under each code were summarised, first within and then across participants. Matrix summaries were reviewed together, with elements of each code compared and organised into key dimensions of experiences, to develop analytic themes. These were discussed and agreed by the research team and with reference to the original data.

Following analysis, integrated interpretation involved comparing the quantitative findings to the qualitative analytic themes to produce consolidated themes. This permitted exploration of the correspondence between the findings and the extent to which they validated or elaborated one another. Qualitative and quantitative findings are presented and discussed together, with respect to the consolidated themes.

### 2.6 Ethical Considerations

Ethical approval was granted by the University of Warwick Humanities and Social Sciences Research Ethics Committee (reference 121/22). The survey was anonymous and began with the presentation of study information followed by consent questions, to which participants were required to agree before proceeding to the main survey. Details of organisations supporting parents of children with disabilities were available at the beginning and end of the survey.

## 3 Results

### 3.1 Participants

Of the 458 prospective participants who visited the first page of the survey, 104 did not complete the consent and eligibility questions, and 37 completed eligibility questions but did not meet the inclusion criteria. Of the remaining 317 participants, 280 answered one or more questions about diagnosis experiences. Of these, 234 (83.6%) provided at least one written response to the open‐ended questions. Sociodemographic information is summarised in Table 1.

**TABLE 1:** Sociodemographic characteristics of participants.

Participant characteristic	*n*	%
Age (years)^a^ (*n* = 280)		
18–24	3	1.1
25–34	78	27.9
35–44	183	65.4
45–54	16	5.7
Missing	0	
Country of residence during pregnancy^a^ (*n* = 280)		
England	246	87.9
Scotland	26	9.3
Wales	8	2.9
Missing	0	
Year of birth of child with Down syndrome^*a*^ (*n* = 280)		
2019	62	22.1
2020	57	20.4
2021	100	35.7
2022	61	21.8
Missing	0	
Sex of child with Down syndrome (*n* = 265)		
Male	151	57.0
Female	114	43.0
Missing	15	
Marital status during pregnancy (*n* = 261)		
Married/civil partnership and living with partner	166	63.6
Living with partner (but not married or in civil partnership)	74	28.4
Single/divorced/separated/widowed/not currently living with partner	21	8.0
Missing	19	
Number of previous children born (*n* = 265)		
None	101	38.1
One	98	37.0
Two	33	12.5
Three or more	33	12.5
Missing	15	
Ethnic group (*n* = 263)		
White	249	94.7
Mixed/multiple	6	2.3
Asian	2	0.8
Black	4	1.5
Arab	2	0.8
Missing	17	
Religious affiliation (*n* = 259)		
No religion	155	59.8
Christian	97	37.5
Muslim	4	1.5
Hindu	1	0.4
Any other religion	2	0.8
Missing	21	
Educational attainment (*n* = 262)		
University degree or higher	176	67.2
No degree	86	32.8
Missing	18	
Financial situation (*n* = 262)		
Living comfortably/doing alright	184	70.2
Just about getting by/find it quite or very difficult	78	29.8
Missing	18	
Financial resources—ability to raise £2000 in 1 week in emergency (*n* = 261)		
Could raise easily/could raise with some sacrifices	158	60.5
Would have to do something drastic to raise/could not raise	103	39.5
Missing	19	

*Note:* Percentages are based on valid responses (*n* shown per item). Missing responses are all eligible participants who did not answer the question.

^**a**^ Indicates questions required for eligibility assessment; hence no missing responses. Other questions were presented at the end of the survey.

There were 163 participants who reported being offered prenatal diagnostic testing, with 56 reporting that they had diagnostic testing and received a prenatal diagnosis.

Quotes are identified by each participant's ID number and whether they received a prenatal or postnatal diagnosis of Down syndrome.

### 3.2 Support With Decisions

**FIGURE 1:**
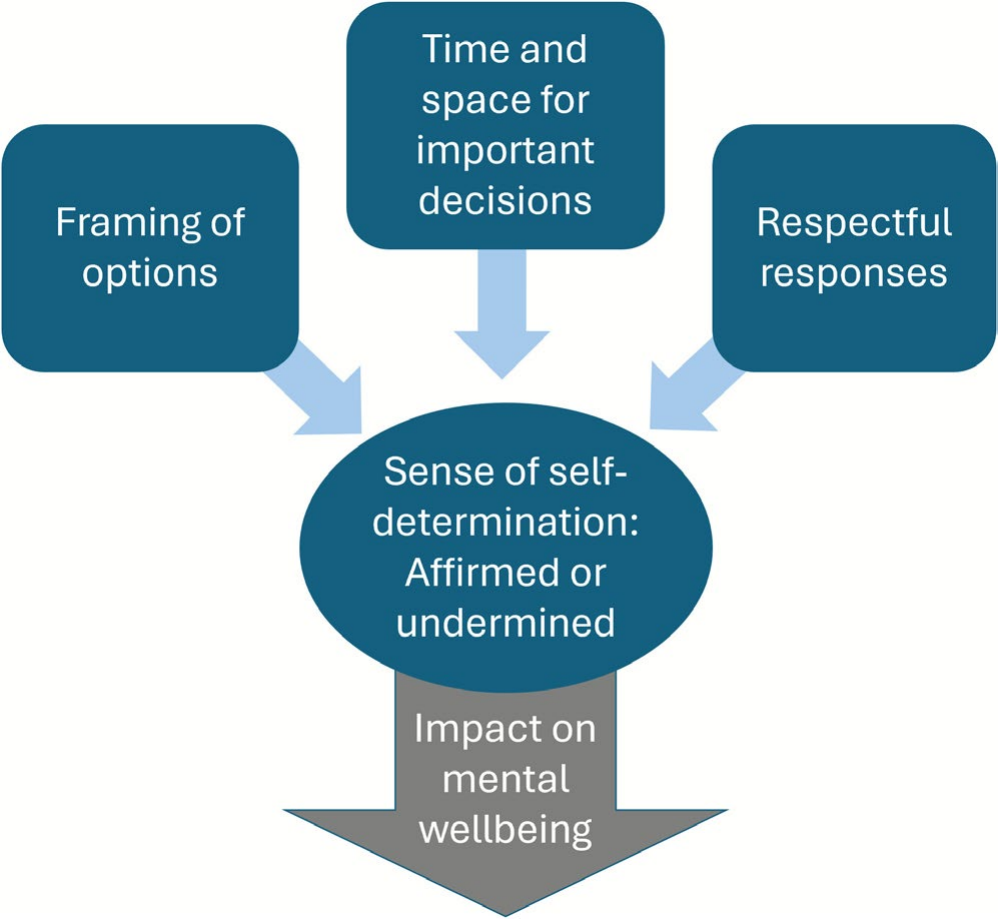
Support with decisions: affirming or undermining.

Findings regarding experiences of support with decisions are organised into three themes: *Framing of options* concerns how information about pregnancy options was conveyed, which for some reflected an emphasis on testing and termination. *Time and space for important decisions* relates to the significance of decisions and support which allowed mothers to make choices at a pace and manner they were comfortable with. *Respectful responses* encompass the responsiveness of interactions to mothers' personal wishes and priorities, and whether these were supported and affirmed, or dismissed and challenged. Together, these aspects contributed to mothers' sense of being supported to reach and realise their personal decisions, or of being constrained or undermined in so doing. This in turn had implications for participants' mental wellbeing: Those who felt they had to defend or reassert their wishes found this distressing and burdensome, while mothers who were positively supported felt validated and more confident in their decisions. A visual representation of these relationships is displayed in Figure 1.

#### 3.2.1 Framing of Options

Almost all participants (97.5%, *n* = 158) who were offered diagnostic testing had understood that this was optional. However, 33.7% (*n* = 55) reported feeling pressured to undergo diagnostic testing, and 19.2% (*n* = 10) of those who received a prenatal diagnosis reported pressure to terminate their pregnancy (Table 2).

**TABLE 2:** Framing of options: Diagnostic testing and pregnancy continuation.

Would you say that you	*n*	%
Understood that having invasive diagnostic testing was optional?^a^ (*n* = 162)		
Yes	158	97.5
No	3	1.9
Not sure	1	0.6
Missing	1	
Got the impression that invasive diagnostic testing was a routine part of antenatal care?^a^ (*n* = 163)		
Yes	39	23.9
No	113	69.3
Not sure	11	6.7
Missing	0	
Felt pressured by healthcare professionals to have invasive diagnostic testing?^a^ (*n* = 163)		
Yes	55	33.7
No	97	59.5
Not sure	11	6.7
Missing	0	
Did you feel pressured by HCPs to make a particular decision?^b^ (*n* = 52)		
Felt pressured to terminate pregnancy	10	19.2
Did not feel pressured	41	78.8
Felt pressured to continue pregnancy	0	0
Not sure	1	1.9
Missing	4	

*Note:* Percentages are based on valid responses (*n* shown per item). Missing responses are all eligible participants who did not answer the question.

^*a*^ Includes only participants who reported that they were directly offered invasive diagnostic testing (*n* = 163).

^b^ Includes only participants who reported receiving a prenatal diagnosis of Down syndrome (*n* = 56).

In written responses, a small number of participants illustrated how professionals supported their decisions by highlighting the voluntary nature of testing, and explaining relevant aspects clearly:

Midwife presented it as an option … She explained the potential risks and benefits and we felt confident in our decision against further testing (PID099, postnatal diagnosis)

However, some participants reported that information shared did not appear neutral, and reflected an emphasis on diagnostic testing and termination. Some participants reported that discussions were framed by implicit or overt negative messages about the prospect of raising a child with Down syndrome:

Every appointment I was offered a termination told that I didn't need the burden of a high needs child as I was still so young and could have “healthy” babies later in life (PID245, postnatal diagnosis)

Some mothers indicated that expectations or assumptions were made about their choices. For example, a small number reported being given unsolicited details about how diagnostic testing or termination was performed, or the necessary conditions for termination—despite making clear this was not relevant to them. Others were explicitly told that termination was the most common choice. One participant highlighted how such expectations entailed failures to provide relevant information and support, contributing to her sense of marginalisation:

I specifically asked if I could be connected to any families who had children with Down syndrome but they were not able to sign post me to any. This made me feel even more alone and that I had made the wrong decision to continue with the pregnancy. It felt like I was the only person who had chosen not to terminate the pregnancy and that normally that's what most people ‘should’ do. (PID260, prenatal diagnosis)

#### 3.2.2 Time and Space for Important Decisions

Most participants (*n* = 75.9%, *n* = 123) reported they had enough opportunity to discuss diagnostic testing with healthcare professionals, and most (78.9%, *n* = 127) had enough time to make their decision about whether to have this test (Table 3).

**TABLE 3:** Time and space for important decisions: diagnostic testing.

Would you say that you	*n*	%
Had enough opportunity to discuss with healthcare professionals whether or not to have invasive diagnostic testing? (*n* = 162)		
Yes	123	75.9
No	34	21.0
Not sure	5	3.1
Missing	1	
Had enough time to decide whether or not to have invasive diagnostic testing? (*n* = 161)		
Yes	127	78.9
No	29	18.0
Not sure	5	3.1
Missing	2	

*Note:* Percentages are based on valid responses (*n* shown per item). Missing responses are all eligible participants who did not answer the question. Includes only participants who reported that they were directly offered invasive diagnostic testing (*n* = 163).

In written responses, several participants highlighted the importance of being able to reach decisions in their own time, and having the opportunity to discuss this with their partner and healthcare team:

I really appreciated being told that I had time to make my decision about whether to continue my pregnancy or not. (PID278, prenatal diagnosis)

However, some participants highlighted how a ‘dismissive’ style of communication seemed to minimise the significance of the choices they faced, and to foreclose meaningful discussion. Others recounted feeling rushed into decisions, particularly when diagnostic testing or termination was offered immediately on receipt of test results or scan findings. This could be an overwhelming experience, constraining mothers' opportunities to meaningfully consider their options:

They pushed for me to have CVS because I was a very high chance with all three chromosome conditions. They offered to do it there and then or the next day, I was in total shock and had no idea what it was all about.” (PID031, prenatal diagnosis)

For a small number of participants, a sense of urgency was compounded by the absence of their partner (sometimes due to Covid‐19 restrictions), leaving them more vulnerable to making hurried decisions:

I had to attend the first appointment with the screening midwife and foetal medicine consultant on my own due to Covid restrictions… My husband was not present and I felt rushed into making a decision about the CVS invasive test … In hindsight, I don't think I would have had the test if my husband was present (PID188, prenatal diagnosis)

#### 3.2.3 Respectful Responses

Most participants (64.3%, *n* = 72) felt their decision to decline diagnostic testing was respected, though 52.7% (*n* = 59) were offered this again after declining, with some being offered several times. Likewise, most participants (77.8%, *n* = 42) reported their decision to continue the pregnancy after prenatal diagnosis was respected, though many (48.9%, *n* = 22) reported that the option of termination was mentioned to them again (Table 4).

**TABLE 4:** Respectful responses: declining diagnostic testing and continuing the pregnancy.

	*n*	%
To what extent did you feel that your decision to decline invasive diagnostic testing was respected?^a^ (*n* = 112)		
Completely/mostly respected	72	64.3
Neither respected nor disrespected	13	11.6
Completely/mostly disrespected	24	21.4
Not sure	3	2.7
Missing	0	
After you told healthcare professionals that you did not wish to have invasive testing, were you offered this again?^a^ (*n* = 112)		
No	49	43.8
Yes, once	8	7.1
Yes, two or three times	30	26.8
Yes, four or more times	21	18.8
Not sure	4	3.6
Missing	0	
To what extent did you feel that your decision to continue your pregnancy was respected?^b^ (*n* = 54)		
Completely/mostly respected	42	77.8
Neither respected nor disrespected	5	9.3
Completely/mostly disrespected	7	13.0
Not sure	0	0
Missing	2	
After you told healthcare professionals that you were continuing your pregnancy, was the possibility of having a termination mentioned again during your maternity care?^c^ (*n* = 45)		
No	22	48.9
Yes, once	4	8.9
Yes, two or three times	12	26.7
Yes, four or more times	5	11.1
Yes, not sure how many times	1	2.2
Not sure	1	2.2
Missing	3	

*Note:* Percentages are based on valid responses (*n* shown per item). Missing responses are all eligible participants who did not answer the question.

^a^ Includes only participants who reported telling healthcare professionals that they did not wish to have invasive diagnostic testing (*n* = 112).

^**b**^ Includes only participants who reported receiving a prenatal diagnosis (*n* = 56).

^c^ Includes only participants who reported receiving a prenatal diagnosis and telling a healthcare professional they were continuing the pregnancy (*n* = 48).

Qualitative findings illustrated how healthcare professionals were experienced as respectful when they took cues from mothers and communicated in ways that centred their wishes. Several mothers appreciated professionals who, being mindful of their intentions, did not mention the option of termination. Similarly, several participants noted that professionals respected their decisions and did not offer diagnostic testing or termination again after they had made their wishes clear:

We had a very good experience in this regard. No one questioned us when we told them our decision and we felt very respected (PID168, postnatal diagnosis)

In contrast, some participants recalled healthcare professionals responding to their decisions with evident surprise. Moreover, professionals were experienced as disrespectful and undermining when they continued to offer or encourage diagnostic testing; participants who had to repeatedly defend their decisions illustrated the emotional impact of this:

the consultant was disrespectful and kept asking me why I didn't want it during my appointment until the point I burst in tears saying I don't want it” (PID206, postnatal diagnosis)

A small number of participants also shared experiences of being repeatedly offered a termination:

Being in labour and the midwife taking your hand and saying ‘I have to tell you that it's not too late to change your mind’. That made a total of 15 times we were asked if we wanted to terminate, the last time being just 20 min before we met him! (PID122, prenatal diagnosis)

Finally, a small number described communication which seemed to suggest they were implicated in their baby's condition, or that it might have been avoided:

In a subsequent appointment with the community midwife, I voiced concerns about my baby's heartbeat, as I had previously suffered a miscarriage. Her reply: ‘well, you knew what you were letting yourself in for’. (PID080, prenatal diagnosis)

#### 3.2.4 Sense of Self‐Determination: Impact on Mental Wellbeing

The preceding themes each contributed to mothers' sense of being supported or undermined in making self‐determined decisions. A number of participants illustrated how this impacted their well‐being during and after pregnancy.

Healthcare professionals were experienced as affirming when they presented information clearly and neutrally, recognised the importance of time for consideration and discussion, and ensured their communication was led by mothers' wishes:

The consultant that I saw and discussed this with was brilliant. He was knowledgeable, professional and invited confidence. He 100% respected my explanation that although nothing would prevent me travelling the road I was on, I just wanted to see a few signposts on the way (PID221, postnatal diagnosis)

However, some mothers who had to defend or reassert their choices, feeling their autonomy was constrained or undermined, described negative consequences for their mental wellbeing:

I feel that my decision to not go ahead with invasive testing was not respected. My decision to continue with my pregnancy was certainly not respected, and the constant pressure to terminate all had an impact on my post‐natal depression and PTSD which followed after birth (PID002, postnatal diagnosis)

Some mothers expressed concern for other expectant parents who may lack the confidence to resist the pressures they experienced. Others described how their trust in professionals and their overall pregnancy experience suffered as a result:

I was asked multiple times if I knew my options (euphemism for abortion) I felt the ones asking us clearly wanted us to terminate our baby which was horrible to have in the back of my mind… that basically they'd rather our baby was dead. It made it difficult to trust the professionals and made the pregnancy harder than it needed to be. (PID008, postnatal diagnosis)

### 3.3 Receiving News About Down Syndrome

**FIGURE 2:**
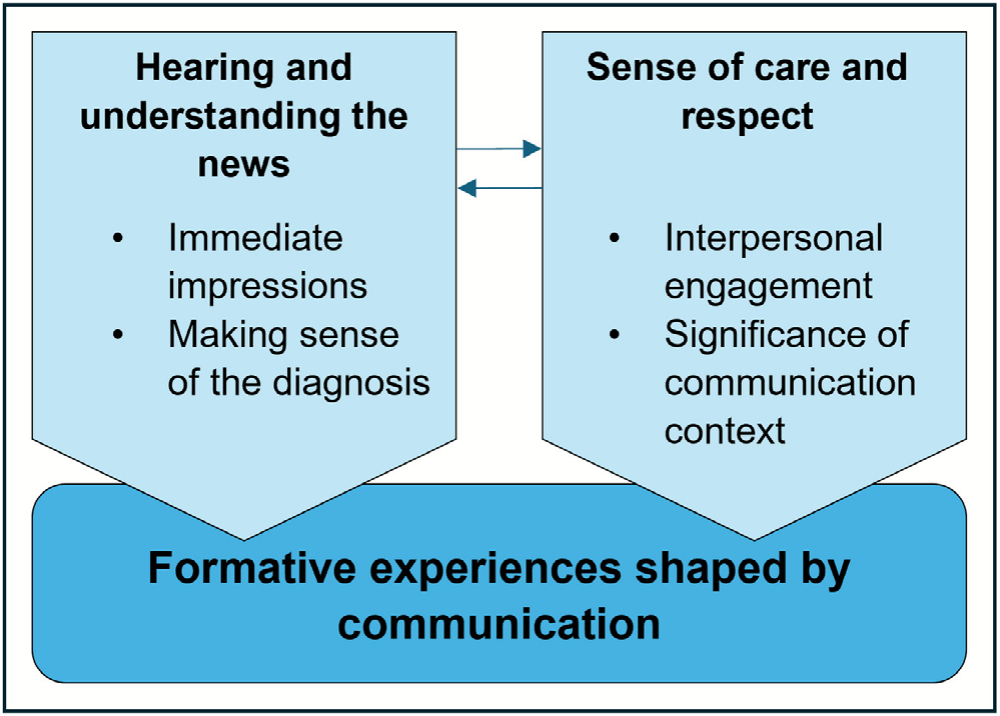
Receiving the news: formative experiences shaped by communication.

Experiences of receiving news about Down syndrome are organised into two overarching themes. Each is underpinned by communication with healthcare professionals, as depicted in Figure 2. Experiences of *hearing and understanding the news* concern professionals' immediate and ongoing communication as it served to characterise Down syndrome. Descriptions of initial language and tone used were prominent, in some cases establishing a sense of consolation or pessimism. Impressions were further shaped by information shared, which some participants felt was disproportionately negative, or insufficient to help them understand the news. *Sense of care and respect* concerned the extent to which interactions with professionals were sensitive, humanising, and empathetic. The context in which communication occurred also contributed to mothers' sense of their needs being respected or sidelined. These two themes were closely related—for example, with mothers' sense of being supported respectfully often corresponding with receipt of balanced and reassuring information about the meaning of the diagnosis.

#### 3.3.1 Hearing and Understanding the News: Immediate Impressions

Participants most commonly reported that their prenatal diagnosis was delivered by a midwife (*n* = 28, 50.0%). Among participants who received postnatal news, most advised this was first shared by a paediatrician or neonatologist (*n* = 115, 55.8%).

Most participants (58.2%, *n* = 153) felt that the prenatal diagnosis or postnatal news of Down syndrome was presented in a neutral way, but 33.5% (*n* = 88) felt it was presented as negative news (Table 5). There was no statistically significant difference in perceptions of negative news presentation between those who received a prenatal and postnatal diagnosis, *χ* ^2^ (1, *N* = 257) = 0.89, *p* = 0.37.

**TABLE 5:** Hearing and understanding the news: Presentation of Down syndrome.

Did you feel that the news was presented to you mainly as	Prenatal diagnosis (*n* = 56)		Postnatal news (*n* = 207)	
	*n*	%	*n*	%
Positive or good news	1	1.8	15	7.2
Neither positive nor negative news	33	58.9	120	58.0
Negative or bad news	22	39.3	66	31.9
Not sure	0	0	6	2.9
Missing	0		17	

*Note:* Percentages are based on valid responses (*n* shown per group). Missing responses are all eligible participants who did not answer the question.

Qualitative findings illustrated diverse experiences of the way news was conveyed, and the impact this had. Participants recalled the positive impact of neutral and transparent communication from professionals who shared practical and encouraging messages:

Consultant was very clear that there was no doubt he definitely had Down syndrome. She didn't present the news negatively and told us life wouldn't be so different. The midwife told me to pick up my baby and cuddle him when I got upset as he was the same baby he was 5 min ago before I got the news which helped. (PID099, postnatal diagnosis)

Mothers expressed appreciation for professionals who were reassuring and celebratory about their child's arrival:

We cannot fault the care we were given when we were told our son may have Down syndrome a few hours after his birth. Every health professional… always congratulated us on the birth of our son before they said anything else. No negative language was ever used and I clearly remember the paediatrician in the hospital reassuring me that first and foremost our son was [child's name] and absolutely beautiful…. (PID219, postnatal diagnosis)

However, a small number of mothers who received a postnatal diagnosis recalled that professionals' positive approach felt incongruent with their own worry or shock:

I actually felt that all medical professionals were almost too positive about it… I felt that no one appreciated that this news came as a shock. (PID200, postnatal diagnosis)

In contrast, among participants who reported a negative presentation of the news, there were recurrent accounts of apologies from healthcare professionals, implying that Down syndrome was an unfavourable outcome:

a lady came to our room and said ‘I’m sorry to say but after checking we believe your baby has Down Syndrome' 'blood tests will confirm' Why were they sorry?? We were happy he was healthy. (PID225, postnatal diagnosis)

Some participants outlined how professionals' initial communication had the power to shape their early perceptions, at a time when they were yet to develop any personal understanding of Down syndrome:

If we had been told about our son having Down Syndrome in a positive, well informed way, we might have started our journey differently. When told by a consultant, when in a vulnerable situation, that it's ‘bad news’, you tend to believe them… until you learn that in fact it's not bad news at all, it's your son and he's amazing. (PID072, postnatal diagnosis)

A number of participants recalled how professionals' manner and non‐verbal communication established a ‘sombre’ mood; more than one participant likened this to an atmosphere befitting a bereavement. Others recalled a lack of positivity and congratulations in professionals' responses to their baby, which felt distinct from other families' experiences:

…I saw the welcome and celebration of other parents, other babies and it was… different. Only one nurse mentioned my son's T21 as a positive or indeed just a part of who he is. (PID221, postnatal diagnosis)

#### 3.3.2 Hearing and Understanding the News: Making Sense of the Diagnosis

As shown in Table 6, most participants felt they received insufficient information about children's abilities, family life, and available support. Many also felt information was lacking about medical aspects of Down syndrome and potential difficulties—though some reported receiving sufficient or too much information about these topics. For all topics, there were no statistically significant differences in perceptions of information sufficiency between those who received a prenatal and postnatal diagnosis.

**TABLE 6:** Hearing and understanding the news: information about specific aspects of Down syndrome.

Do you feel you received the right amount of information about the following topics	Prenatal diagnosis		Postnatal news	
	*n*	%	*n*	%
Medical or physical aspects of Down syndrome (prenatal: *n* = 55; postnatal: *n* = 203)				
Received too much information	6	10.9	23	11.3
Received the right amount of information	22	40.0	74	36.5
Received too little information	23	41.8	85	41.9
Not sure	4	7.3	21	10.3
Missing	1		21	
Difficulties that children/people with Down syndrome may have (besides medical difficulties) (prenatal: *n* = 55; postnatal: *n* = 203)				
Received too much information	12	21.8	41	20.2
Received the right amount of information	21	38.2	59	29.1
Received too little information	19	34.5	79	38.9
Not sure	3	5.5	24	11.8
Missing	1		21	
Abilities that children/people with Down syndrome may have (prenatal: *n* = 54; postnatal: *n* = 205)				
Received too much information	4	7.4	15	7.3
Received the right amount of information	17	31.5	46	22.4
Received too little information	30	55.6	121	59.0
Not sure	3	5.6	23	11.2
Missing	2		19	
What everyday life may be like for families raising a child with Down syndrome (prenatal: *n* = 54; postnatal: *n* = 205)				
Received too much information	3	5.6	10	4.9
Received the right amount of information	14	25.9	41	20.0
Received too little information	34	63.0	137	66.8
Not sure	3	5.6	17	8.3
Missing	2		21	
Support available for families raising a child with Down syndrome (prenatal: *n* = 55; postnatal: *n* = 206)				
Received too much information	0	0	3	1.5
Received the right amount of information	17	30.9	48	23.3
Received too little information	38	69.1	144	69.9
Not sure	0	0	11	5.3
Missing	1		18	

*Note:* Percentages are based on valid responses (*n* shown per item). Missing responses are all eligible participants who did not answer the question.

Fewer than half the participants felt that information received about Down syndrome at the time of diagnosis was balanced (40.8%, *n* = 97), accurate (40.1%, *n* = 87), or up to date (33.8%, *n* = 74), while 50.5% (*n* = 109) found it relevant, and 57.4% (*n* = 132) found it easy to understand (Table 7). There were no statistically significant differences in perceptions of information quality between those who received a prenatal and postnatal diagnosis.

**TABLE 7:** Hearing and understanding the news: Quality of information received.

Would you say the information you were given about Down syndrome was	Prenatal diagnosis		Postnatal news	
	*n*	%	*n*	%
Balanced (prenatal: *n* = 50; postnatal: *n* = 188)				
Yes	23	46.0	74	39.4
No	25	50.0	94	50.0
Not sure	2	4.0	20	10.6
Missing	6		36	
Relevant to you (prenatal: *n* = 46; postnatal: *n* = 170)				
Yes	27	58.7	82	48.2
No	14	30.4	61	35.9
Not sure	5	10.9	27	15.9
Missing	10		54	
Accurate (prenatal: *n* = 46; postnatal: *n* = 171)				
Yes	23	50.0	64	37.4
No	20	43.5	75	43.9
Not sure	3	6.5	32	18.7
Missing	10		53	
Up to date (prenatal: *n* = 46; postnatal *n* = 173)				
Yes	19	41.3	55	31.8
No	22	47.8	90	52.0
Not sure	5	10.9	28	16.2
Missing	10		51	
Easy to understand (prenatal: *n* = 52; postnatal *n* = 178)				
Yes	36	69.2	96	53.9
No	13	25.0	66	37.1
Not sure	3	5.8	16	9.0
Missing	4		46	

*Note:* Percentages are based on valid responses (*n* shown per item). Missing responses are all eligible participants who did not answer the question.

In qualitative responses, several participants expressed unmet needs for practical information about caring for their child, supporting their development, and navigating relevant services. Some participants who received the news postnatally reported that delays in information provision—due to staff unavailability, or information being withheld until the diagnosis was confirmed—contributed to their worry. Others reported that hospital literature was outdated or unavailable, with professionals instead printing information from the internet, or telling parents to search online themselves.

Some participants described the emotional and practical impacts of receiving insufficient information about Down syndrome, leaving them feeling unsupported, isolated, or unprepared:

…the hospital gave medical overviews but nothing about day to day life or helped us as a family prepare in any way. (PID071, prenatal diagnosis)

Several participants recounted a predominance of negative information, or a focus on medical issues. Some described information suggesting that hardship and difference were inherent to Down syndrome, or which conveyed low expectations about their child's prospects:

…But no positive information given. When asked if he'd be able to speak, walk, etc. we were told unable to tell so early. I feel we should have been told. Yes most people with Down syndrome do do those things and more… (PID194, postnatal diagnosis)

In contrast, participants highlighted their appreciation of simple, practical advice which normalised their child's needs, rather than contributing to a sense of difference. Others recalled the reassurance they received through opportunities to connect with other parents, and the provision of realistic, meaningful information:

The care and approach given by the medical team… was balanced and neutral in terms of the outlook for our child once we received a positive diagnosis ‐ our consultant presented an informed and balanced picture of what life would likely be like and indicated a spectrum of ability and capability of babies with Down syndrome. Their indication was that, although people with Down syndrome can fall at extreme ends of the spectrum, the reality was that our baby would go on to lead a happy and fulfilled life. (PID019, prenatal diagnosis)

Meanwhile, a small number of participants described that receiving too much information at once could be overwhelming:

At the time I found it hard to process so anymore information I would not have coped with nor wanted. (PID035, postnatal diagnosis)

#### 3.3.3 Sense of Care and Respect: Interpersonal Engagement

Beyond information about Down syndrome, qualitative findings demonstrated the importance of relational aspects of care. Several participants recounted impactful experiences of professionals showing kindness and sensitivity, often through simple acts which demonstrated genuine consideration for parents' emotional and informational needs:

The positive was the healthcare support lady who telephoned us on our way back to the car who was unhappy with the doctor's reaction and found an information pack from a neighbouring trust in the office to give to us. She went out of her way from that point onwards to be supportive and for that, we will be eternally grateful (PID080, prenatal diagnosis)

However, some participants recalled experiences of professionals adopting a detached or avoidant approach when sharing the news. Several described communication as blunt or cold, and interactions marked by a lack of empathy or humanity. Several participants highlighted that language used to describe their baby, either verbally or in written records, was upsetting or offensive. Further, some felt their baby was treated as a spectacle, describing professionals ‘feature spotting’. Often, a clinical approach accompanied a failure to recognise mothers' needs for information. The following excerpt illustrates several such aspects, and their lasting impact:

…one doctor just stared at me the entire time without saying a single word. The other doctor spent what felt like a very long time describing various features of my child without explaining why he was telling me these things. He used the words deformities and abnormalities making me wonder if my child had missing limbs, cleft palate. etc.… Then finally looking very awkward he said they suspected Down syndrome and apologised…reinforcing what a bad thing he thought it was. He then left without providing ANY information on Down syndrome… It was awful and I will never forget it. (PID181, postnatal diagnosis)

#### 3.3.4 Sense of Care and Respect: Significance of Communication Context

Aspects of the communication context were also experienced as demonstrating sensitivity or insensitivity to mothers' emotional needs. Quantitative findings showed that most participants who received postnatal news were given this in the presence of their partner, where applicable (*n* = 133, 77.8%). However, several mothers who received a postnatal diagnosis recounted the indignity of this being shared on an open ward:

I was told in the middle of NICU, without my husband present. Completely insensitive and no privacy. (PID124, postnatal diagnosis)

Participants also highlighted the significance of the timing of postnatal news. While some felt that professionals had not been forthright in disclosing their suspicions, others felt that the news had been shared too immediately after birth, which impacted their opportunity to bond with their baby:

I was told within minutes of giving birth that my baby may have Down syndrome. I wish they could have waited at least an hour before telling me this, so I could have enjoyed holding my baby without worrying about what this diagnosis could mean for his future. She ruined that moment of bliss and I can never get that back. (PID117, postnatal diagnosis)

## 4 Discussion

This study drew on a large sample to explore the range of experiences and perceptions of mothers who received a Down syndrome diagnosis within recent NHS maternity care. Our findings highlight the critical role of healthcare professionals' communication and support in shaping maternal experiences of this significant life event. Quantitative findings indicated that negative care experiences were typically in the minority, but a notable exception was the provision of information about Down syndrome, which most participants felt was insufficient, imbalanced, and outdated. Qualitative findings demonstrated the positive impact of sensitive, supportive approaches on mothers' emotional adjustment and coping with both prenatal decision‐making and receipt of the diagnosis. Conversely, the communication of assumptions—such as that parents will choose testing and termination, or that news of the diagnosis is inherently negative—was experienced as undermining and could impact trust in professionals. A lack of affirmation for those continuing the pregnancy with or without prenatal diagnosis; a focus on deficits; or a failure to provide realistic, meaningful information contributed to feelings of isolation and invalidation, compounding the emotional impact of the diagnosis.

This study adds to existing evidence that communication of the Down syndrome diagnosis is highly memorable and impacts parental perceptions and coping (Kammes et al. 2022; May et al. 2020; Nelson Goff et al. 2013). It extends prior research by focusing on the NHS context, where the national screening programme and broad uptake of prenatal testing and termination may influence healthcare interactions. In contrast to recent research from the USA (Meredith et al. 2024, 2025), most mothers in this study felt the diagnosis was presented neutrally. However, our findings also illustrated how language and non‐verbal communication could colour early experiences, setting a tone of misfortune and pity. Like studies from North America and Europe (e.g., Artal et al. 2025; Asgarova 2019; Crombag et al. 2020), we found that some mothers continue to report healthcare providers emphasising testing and termination, with one third of the present participants having felt pressured to undergo diagnostic testing. This study contributes additional evidence about the emotional impact of such experiences. Having to repeatedly defend or reassert their reproductive decisions was emotionally burdensome for mothers, while interactions implying their choices deviated from normative expectations contributed to feelings of marginalisation.

A key potential benefit of prenatal testing is the possibility it offers for emotional and practical preparation for a child with Down syndrome (Michie 2020). However, our findings align with previous research indicating that many mothers did not receive sufficient information to help them understand and prepare for parenthood (e.g., Buyukavci et al. 2019; Reed and Berrier 2017). While most participants felt that information was not balanced, many also felt there was insufficient information on all topics, including medical aspects of Down syndrome, and other potential difficulties. No significant differences in perceptions of information received were identified between participants who received a prenatal and postnatal diagnosis, reflecting that these groups are similarly affected by unmet informational needs. Participants valued timely, clear, and realistic information, and signposting to support services, a lack of which could exacerbate feelings of isolation. As in previous research (Van Riper and Choi 2011), mothers highlighted the importance of realistic, reassuring narratives, which fostered a sense of possibility and normality (Clark et al. 2020). In contrast, a focus on potential deficits and the absence of positive or congratulatory messages contributed to a sense of difference or ‘otherness,’ in line with previous findings (Lalvani 2011; Sooben 2010).

### 4.1 Limitations

The sample has limitations concerning its representativeness: relative to the wider British population, women with lower educational attainment and from non‐white ethnic backgrounds are underrepresented. Recruitment was conducted through charities, which may have resulted in a sample representing those with greater access to support and resources. The online survey method can also attract fraudulent responses, though the present study was considered at low risk since it involved no financial incentives, was not indexed on search engines, and recruited participants via closed networks. We did not include women who terminated their pregnancy, despite this being the most common outcome following prenatal diagnosis of Down syndrome in Great Britain (NHS England 2024). Further, some responses may have been affected by recall bias, potentially influencing the accuracy of reported experiences. Finally, certain survey questions received a low number of responses (e.g., Tables 6 and 7). This is partly explained by an initial technical issue in the survey setup which meant that these questions were inadvertently not shown to all participants. Missing responses may also have been due to participant fatigue, since questions presented earlier were less affected, and some participants did not reach the end of the survey. These factors may have limited the completeness of the findings and resulted in a sample representing more highly engaged participants.

### 4.2 Implications

These findings highlight the importance of family‐centred communication in delivering a Down syndrome diagnosis. Healthcare professionals can support parents by providing sufficient time and opportunities to consider information, and recognising and responding to individual priorities. In the prenatal context, support for mothers' autonomy—particularly maintaining an open and unassuming approach—significantly contributes to emotional wellbeing.

Timing of communication was an important consideration postnatally, with the findings highlighting the need to protect early opportunities for parent–child bonding, while avoiding delays in information‐sharing that could exacerbate anxiety. Wherever possible, postnatal news should be delivered in a private setting to both parents together (see also Gori et al. 2023; Skotko et al. 2009; Van Riper and Choi 2011), which preserves dignity and demonstrates sensitivity to the ‘enormity’ of the news (Hodgson et al. 2016).

Many participants expressed unmet needs for practical, positive and realistic information about their child's development and available support. The current findings underscore the importance of tailoring information provision to each family; for example, some mothers felt overwhelmed by receiving too much information at once. Professionals require knowledge of contemporary resources and services, enabling them to offer relevant guidance at the appropriate time. Many of these implications correspond to existing international guidelines about communicating a Down syndrome diagnosis (e.g., Gori et al. 2023; Sheets et al. 2011; Skotko et al. 2009), which demonstrate their relevance to the British context, and the potential benefits of wider dissemination among NHS professionals.

### 4.3 Further Research

Future research could explore factors shaping healthcare professionals' approaches to communication, information‐sharing, and parental support, including the respective contributions of training, professional guidelines, and peer learning. This could help to identify where further efforts and resources may be targeted to improve parental experiences. Such research may offer additional insights into the emotional impact of this work on professionals, and how they can be better supported in this vital role.

In addition, longitudinal studies could provide valuable information about how early healthcare experiences may affect parental adjustment and wellbeing in the longer term. This could help to clarify the implications of maternity care experiences, and opportunities to make a lasting positive difference.

## 5 Conclusions

Expectant and new mothers require high‐quality information, sensitive and open communication, and personalised support in relation to their child's Down syndrome diagnosis. While many participants described positive care experiences that met these needs, others reported negative assumptions, a sense of disrespect for their values and choices, and insufficient or imbalanced information received. Some mothers felt their autonomy was undermined and reported negative consequences for their mental wellbeing. These findings reiterate the vital role of healthcare professionals in helping to validate reproductive decisions, support the transition to parenthood, and reduce isolation and stigmatisation. With enhanced training, support and resources, professionals would be better able to meet these critical needs.

## Author Contributions

**Tamar Rutter:** conceptualisation, methodology, data curation, formal analysis, writing – original draft. **Chris Stinton:** conceptualisation, methodology, supervision, writing – review and editing. **Nicola Enoch:** conceptualisation, supervision, writing – review and editing. **Samantha Flynn:** methodology, supervision, writing – review and editing. **Matthew Randell:** formal analysis, writing – review and editing. **Richard Hastings:** conceptualisation, methodology, supervision, writing – review and editing.

## Funding

This work was supported by the Economic and Social Research Council, ES/P000771/1 and Down Syndrome UK.

## Conflicts of Interest

This research is associated with the PhD studentship awarded to T.R. and supervised by C.S. and R.H. The studentship has been partially funded by Down Syndrome UK, a national charity supporting families and their children with Down syndrome. N.E. is the founder and chief executive of Down Syndrome UK. The remaining authors have no conflicts of interest to declare.

## Supporting information


**Data S1:** Supporting Information.

## Data Availability

The data that support the findings of this study are available on request from the corresponding author. The data are not publicly available due to privacy or ethical restrictions.
