# Supplementary material for: Down Syndrome in British Maternity Care: Mothers' Experiences of Prenatal Testing and Receiving a Prenatal or Postnatal Diagnosis
Source: J Appl Res Intellect Disabil. 2025 Dec 8;38(6):e70160. doi: 10.1111/jar.70160 (PMC12683471; doi:10.1111/jar.70160)
Supplement: Supplementary file 1 — Data S1: Supporting Information. [file JAR-38-e70160-s001.docx]

**Supplementary Information A: Survey questions**

Contents

[SECTION 1: Introduction 2](#_Toc194934102)

[SECTION 2: Participant Information Leaflet and Consent Form 3](#_Toc194934103)

[SECTION 3: Eligibility questions 4](#_Toc194934104)

[SECTION 4: Questions about initial screening offer (*all respondents)* 6](#_Toc194934105)

[SECTION 5: Questions about screening choice (*all respondents*) 8](#_Toc194934106)

[SECTION 6: Questions about declining screening (*only displayed to respondents who were offered and declined screening)* 9](#_Toc194934107)

[SECTION 7: Questions about screening results *(only displayed to respondents who had the combined or quadruple screening test)* 10](#_Toc194934108)

[SECTION 8: Questions about NIPT offer (*all respondents*) 13](#_Toc194934109)

[SECTION 9: Questions about declining NIPT (*only displayed to respondents who were offered NIPT and declined it)* 15](#_Toc194934110)

[SECTION 10: Questions about NIPT results (*only displayed to respondents who had NIPT*) 16](#_Toc194934111)

[SECTION 11: Questions about offer of prenatal diagnostic testing (*only displayed to respondents who received higher chance combined/quadruple screen or higher chance/ inconclusive NIPT result)* 18](#_Toc194934112)

[SECTION 12: Questions about declining prenatal diagnostic testing (*only displayed to respondents who were offered and did not have diagnostic testing)* 20](#_Toc194934113)

[SECTION 13: Questions about receiving prenatal diagnosis *(only displayed to respondents who received prenatal diagnosis)* 21](#_Toc194934114)

[SECTION 14: Questions about discussion of termination *(only displayed to respondents who received prenatal diagnosis)* 24](#_Toc194934115)

[SECTION 15: Questions about receiving postnatal news (*only displayed to respondents who did not receive prenatal diagnosis*) 26](#_Toc194934116)

[SECTION 16: Questions about overall maternity experiences (*All respondents)* 29](#_Toc194934117)

[SECTION 17: Sociodemographic questions (*all respondents*) 31](#_Toc194934118)

[END OF SURVEY message (*all respondents)* 33](#_Toc194934119)

*NB. Contents (above) and section headings not shown to participants. All questions permit only one response to be selected, unless otherwise stated.*

# SECTION 1: Introduction

**Down syndrome in maternity care: Mothers’ experiences of testing and diagnosis**

Thank you for your interest in taking part in this study, which is an online survey about the maternity care experiences of mothers of young children with Down syndrome.

The survey will take 20 – 30 minutes to complete. You can leave the survey at any time and your responses will be recorded. If you access the survey again using the same device and the same internet browser (without deleting your internet browsing history), you will be able to pick up where you left off and continue completing the survey later, provided you do so within 2 weeks of your last response.

The next page will display the participant information leaflet, which contains important information about the study. This information is also available to view or download on the study webpage (*link*). After this information, there will be some questions to confirm that you consent and are eligible to participate.

 Please click on the arrow below to proceed to the participant information leaflet.

# SECTION 2: Participant Information Leaflet and Consent Form

*(Participant Information Leaflet inserted here)*

*(Consent Form inserted here)*

# SECTION 3: Eligibility questions

We are collecting information from biological mothers of children with Down syndrome who were born in 2019 or later and are now at least 2 months old. We are collecting information from mothers who were living in England, Scotland or Wales during their pregnancy, and who are at least 18 years old.

**These first questions are to check if you are eligible to participate in this study.**

1. Are you the biological mother of a child with Down syndrome?

By ‘biological mother’ we mean the parent who has carried the pregnancy and given birth.

- Yes
- No → *to exit message*

1. When was your child with Down syndrome born?
   1. 2018 or earlier→ *to exit message*
   2. 2019
   3. 2020
   4. 2021
   5. 2022

2b. (*if ‘2022’ is selected in previous question*) How old is your child with Down syndrome?

1. 2 months old or older
2. Under 2 months old*→ to exit message*
3. Which country of the UK were you living in while you were pregnant with your child with Down syndrome?

(If you were living in more than one country, please select the country where you received the majority of your maternity care)

- 1. England
  2. Scotland
  3. Wales
  4. I was not living in England, Scotland or Wales while pregnant with my child with Down syndrome → *to exit message*

1. How old are you?
   1. Under 18 years old→ *to exit message*
   2. 18 to 24 years old
   3. 25 to 29 years old
   4. 30 to 34 years old
   5. 35 to 39 years old
   6. 40 to 44 years old
   7. 45 to 49 years old
   8. 50 to 54 years old
   9. 55 years old and over

*Exit message displayed to ineligible respondents:*

“Thank you very much for your interest in participating in this survey, but at present we are only collecting responses from biological mothers of children with Down syndrome who were born in 2019 or later and are now at least 2 months old. We are only collecting responses from mothers who were living in England, Scotland or Wales during this pregnancy and who are aged 18 and over.

If you would like to find out more about this research, please see the information sheet on this website: (*link to study webpage*), or for further information you can contact [removed for blind review].”

# SECTION 4: Questions about initial screening offer (*all respondents)*

This survey will ask about each stage in the prenatal testing pathway in turn (depending on your experiences). When responding, please try to answer only about your experiences for that particular stage.

**The first stage of screening for Down syndrome is offered to all expectant mothers. This is the ‘combined test’- which is an ultrasound scan and a blood test - or (if later in the pregnancy), the ‘quadruple test’ - which is a blood test only.**

**These questions are about your experiences of being offered the combined or quadruple screening test.**

**If you have been pregnant more than once, when you are responding to these questions please think only about your experiences while you were pregnant with your baby with Down syndrome, who you told us was born in _ [***autofill year of birth from response to question 2*].

1. Which healthcare professional(s) discussed the possibility of having screening tests for Down syndrome with you?

Please select the healthcare professional(s) that talked to you about screening *before* you chose whether or not to have it.

Select all that apply

- 1. Midwife >*to question 5b*
  2. GP
  3. Obstetrician or Gynaecologist (Doctor who specializes in pregnancy/ women’s reproductive health)
  4. Genetic counsellor
  5. Other Doctor
  6. Nurse (e.g., practice nurse at GP surgery)
  7. None
  8. Not sure
  9. Other (please state):

5b. (*Display only if ‘Midwife’ is selected in previous question)* Which kind of midwife discussed screening tests for Down syndrome with you?

Select all that apply

1. Community midwife
2. Hospital midwife
3. Screening midwife
4. Private / Independent midwife
5. Other
6. Not sure
7. Before you were asked if you wanted to have screening tests for Down syndrome, were you:
   1. Given written information about screening tests for Down syndrome
   2. Signposted to online information about screening tests for Down syndrome
   3. Neither of these
   4. Not sure

(*Possible to select both* ***a*** *and* ***b***)

6b. (*Display only if ‘written information’ is selected in previous question*)

Which written information were you given about screening tests for Down syndrome?

1. The NHS booklet ‘Screening tests for you and your baby’
2. Other written information about screening tests
3. Not sure

(*Possible to select both ‘a’ and ‘b’*)

1. Thinking about the way that healthcare professionals supported you to consider whether or not to have screening tests for Down syndrome -

Would you say that:

|  | Yes | No | Not sure |
| --- | --- | --- | --- |
| 1. You understood that screening tests for Down syndrome were optional? |  |  |  |
| 1. You got the impression that screening tests for Down syndrome were a routine part of antenatal care? |  |  |  |
| 1. You had enough time to decide whether or not to have screening tests for Down syndrome? |  |  |  |
| 1. You had enough opportunity to discuss with healthcare professional(s) whether or not to have screening tests for Down syndrome? |  |  |  |
| 1. You felt pressured by healthcare professionals to have screening tests for Down syndrome? |  |  |  |
| 1. You ‘went along with’ having screening tests, without giving them much thought? |  |  |  |

1. Would you like to share anything else about the information, advice or support that you received from healthcare professionals in relation to your choice whether or not to have screening tests for Down syndrome? If so, please write it in this box.

# SECTION 5: Questions about screening choice (*all respondents*)

Did you have either the combined or quadruple screening test for Down syndrome?

Yes >*skip* *to section 7*

No

Not sure

(*Displayed only if answered ‘no’ to previous question:*) Were you offered the combined or quadruple screening test for Down syndrome?

Yes, I was offered a screening test, but I told healthcare professionals I did not wish to have it *>to section 6*

No, I was not offered a screening test *>skip to section 8*

Not sure *>skip to section 8*

# SECTION 6: Questions about declining screening (*only displayed to respondents who were offered and declined screening)*

After you had told a healthcare professional that you did not wish to have the combined or quadruple screening test for Down syndrome, were you offered this again?

Yes > *to question 11b*

No

Not sure

11b. (*Only displayed if answered ‘yes’ to previous question)* How many additional times were you offered the combined or quadruple screening test after telling healthcare professionals that you did not wish to have it?

Once

2 or 3 times

4 or more times

Not sure

To what extent did you feel that your decision not to have the combined or quadruple screening test was respected by healthcare professionals?

My decision was…

Completely respected

Mostly respected

Neither respected nor disrespected

Mostly disrespected

Completely disrespected

Not sure

# SECTION 7: Questions about screening results *(only displayed to respondents who had the combined or quadruple screening test)*

**These questions are about your experiences of receiving the result of your combined or quadruple screening test.**

What was the result of your combined or quadruple screening test?

The test indicated a higher chance of baby having Down syndrome

- 1. The test indicated a lower chance of baby having Down syndrome
  2. Not sure

13b. Please advise, if you recall, the probability you were given of your baby having Down syndrome based on your combined or quadruple screening.

(e.g., 1 in 100, etc.)

1 in _____

1. How was the screening result first shared with you?
   1. In person >*to question 15*
   2. By telephone *>to question 16*
   3. By post/email *>to question 16*
   4. Other - Please specify: *>to question 16*
2. *(Displayed only if ‘In person’ is selected in previous question)* Was your partner present when the result was shared with you?
   1. Yes
   2. No
   3. Not sure
   4. Not applicable
3. Which healthcare professional first shared the result of your combined or quadruple screening test with you?
4. Midwife
5. GP
6. Obstetrician or Gynaecologist (Doctor who specializes in pregnancy/ women’s reproductive health)
7. Genetic counsellor
8. Other Doctor
9. Nurse (e.g., practice nurse at GP surgery)
10. Receptionist / secretary / administrator
11. Not sure
12. Other (please specify):

16b. (*Displayed only if ‘midwife’ is selected in previous question*) Which kind of midwife shared the result of your combined or quadruple screening test with you?

a. Community midwife

b. Hospital midwife

c. Screening midwife

d. Private / Independent midwife

e. Other

f. Not sure

Did you feel that your combined or quadruple screening test result was presented to you as:

Good / positive news

Neither good nor bad news

Bad / negative news

Not sure

1. When you were given your combined or quadruple screening test result, were you:
   1. Given written information about Down syndrome
   2. Signposted to online information about Down syndrome
   3. Neither of these
   4. Not sure

(*Possible to select both* ***a*** *and* ***b***)

18b. When you were given your combined or quadruple screening test result, do you feel you were given:

1. Too much information about Down syndrome
2. The right amount of information about Down syndrome
3. Not enough information about Down syndrome
4. Not sure
5. When you were given your combined or quadruple screening test result, did you receive information or support from any of the following organisations:

Select all that apply

- 1. Down’s Syndrome Association (DSA)
  2. Down’s Syndrome Scotland (DSS)
  3. Positive About Down Syndrome (PADS)
  4. Antenatal Results and Choices (ARC)
  5. Local support group
  6. Other – please specify: ______________
  7. None of these
  8. Not applicable
  9. Not sure

(*Possible to select any combination of* ***a, b, c, d, e,*** *and* ***f***)

19b. (*Only displayed to respondents who selected at least one of the options a – f in question 19*) How helpful did you find the information or support you received from each organisation?

(*Only organisations selected in previous question are displayed)*

|  | Very helpful | Somewhat helpful | Not helpful | Not sure |
| --- | --- | --- | --- | --- |
| 1. Down Syndrome Association (DSA) |  |  |  |  |
| 1. Down’s Syndrome Scotland (DSS) |  |  |  |  |
| 1. Positive About Down Syndrome (PADS |  |  |  |  |
| 1. Antenatal Results and Choices (ARC) |  |  |  |  |
| 1. Local support group ____________ |  |  |  |  |
| 1. (*Other:)* |  |  |  |  |

Would you like to share anything else about your experiences of being given your combined or quadruple screening test result? If so, please write it in this box.

# SECTION 8: Questions about NIPT offer (*all respondents*)

**A new step in the pathway of prenatal testing for Down syndrome is NIPT, or Non-Invasive Prenatal Testing. NIPT analyses DNA from the baby which is found in the pregnant mother’s blood.**

**NIPT was introduced into NHS maternity services in Great Britain between 2018 and 2021, as an offer to expectant parents if the combined or quadruple screening test has found their baby to have a higher chance of having Down syndrome. NIPT is sometimes called cell-free DNA (cfDNA) testing or DNA testing. It may also be referred to by brand names such as Harmony, Panorama or IONA.**

Still thinking only about your experiences while you were pregnant with your baby with Down syndrome who was born in __ [*auto-fill birth year*] –

1. Were you offered Non-Invasive Prenatal Testing (NIPT) for Down syndrome as part of your **NHS maternity healthcare**?

For this question, please only tell us whether you were offered NIPT in the NHS.

- 1. Yes
  2. No
  3. Not sure

Did you access **privately-funded** (non-NHS) maternity healthcare in order to have NIPT for Down syndrome?

Yes

No

1. *(Displayed only if answered ‘yes’ to either question 21 or 22)* Thinking about the way that healthcare professionals supported you to consider whether or not to have NIPT for Down syndrome –

Would you say that:

|  | Yes | No | Not sure |
| --- | --- | --- | --- |
| 1. You understood that NIPT cannot tell you for certain if your baby has Down syndrome? |  |  |  |
| 1. You understood that having NIPT for Down syndrome was optional? |  |  |  |
| 1. You got the impression that NIPT was a routine part of antenatal care? |  |  |  |
| 1. You had enough time to decide whether or not to have NIPT for Down syndrome? |  |  |  |
| 1. You had enough opportunity to discuss with healthcare professional(s) whether or not to have NIPT for Down syndrome? |  |  |  |
| 1. You felt pressured by healthcare professionals to have NIPT for Down syndrome? |  |  |  |
| 1. You ‘went along with’ having NIPT, without giving it much thought? |  |  |  |

*(Displayed only to respondents who answered ‘Yes’ to question 21 and ‘No’ to question 22)* Did you have NIPT for Down syndrome?

Yes >*to section 10*

No >*to section 9*

Not sure > *to section 11*

# SECTION 9: Questions about declining NIPT (*only displayed to respondents who were offered NIPT and declined it)*

After you had told a healthcare professional that you did not wish to have NIPT for Down syndrome, were you offered NIPT again?

Yes

No

Not sure

25b. (*Displayed only if answered ‘Yes’ to previous question*) How many additional times were you offered NIPT after telling healthcare professionals that you did not wish to have it?

Once

2 or 3 times

4 or more times

Not sure

To what extent did you feel that your decision not to have NIPT was respected by healthcare professionals?

My decision was…

Completely respected

Mostly respected

Neither respected nor disrespected

Mostly disrespected

Completely disrespected

Not sure

1. Would you like to share anything else about the care you received in relation to your choice not to have NIPT? If so, please write it in this box.

# SECTION 10: Questions about NIPT results (*only displayed to respondents who had NIPT*)

**These questions are about your experiences of receiving the results of your Non-Invasive Prenatal Test (NIPT).**

What was your NIPT result?

Higher chance (see further details)^1^

Lower chance (see further details)^2^

Inconclusive

Not sure

*^12^When respondents hover over the words “see further details”, the following text will appear:*

^1^ “This may also have been referred to as high chance, increased likelihood, or a positive result for Down syndrome”

^2^ “This may also have been referred to as low chance, reduced likelihood, or a negative result for Down syndrome”

How was your NIPT result first shared with you?

- 1. In person >*to question 30*
  2. By telephone *>to question 31*
  3. By post/email *>* *to question 31*
  4. Other (please specify) *>* *to question 31*

(*If ‘In person’ is selected*) Was your partner present when the NIPT result was shared with you?

Yes

No

Not applicable

Not sure

Which healthcare professional first shared your NIPT result with you?

1. Midwife >*to question 31b*
2. GP
3. Obstetrician or Gynaecologist (Doctor who specializes in pregnancy/ women’s reproductive health)
4. Genetic counsellor
5. Other Doctor
6. Nurse (e.g., practice nurse at GP surgery)
7. Receptionist / secretary / administrator
8. Not sure
9. Other (please specify):

31b. (*Displayed only* *if ‘midwife’ is selected in previous question*) Which kind of midwife shared the NIPT result with you?

- 1. Community midwife
  2. Hospital midwife
  3. Screening midwife
  4. Private / Independent midwife
  5. Other
  6. Not sure

Did you feel that your NIPT result was presented to you as:

Good / positive news

Neither good nor bad news

Bad / negative news

Not sure

Would you like to share anything else about your experiences of being given your NIPT result? If so, please write it in this box

# SECTION 11: Questions about offer of prenatal diagnostic testing (*only displayed to respondents who received higher chance combined/quadruple screen or higher chance/ inconclusive NIPT result)*

**After receiving a higher chance result from the combined or quadruple screening test and/or NIPT, expectant parents are offered an invasive diagnostic test (amniocentesis or chorionic villus sampling (CVS)) which involves the collection of cells through a needle inserted into the womb. This test is presently the only way to confirm before birth whether a baby has Down syndrome.**

1. Was the possibility of having invasive diagnostic testing mentioned during your antenatal care?
   1. Yes, this was mentioned during a conversation with healthcare professionals
   2. Yes, this was mentioned in a letter / email from healthcare professionals
   3. I mentioned this myself since I was aware of it
   4. No, this was never mentioned to me > *skip to section 15*
   5. Not sure

(*Possible to select both* ***a*** *and* ***b***)

1. Did a healthcare professional directly ask you if you wanted to have invasive diagnostic testing?
   1. Yes > *to question 35b*
   2. No
   3. Not sure

35b. (*Displayed only if responded ‘yes’ to question 35)* When did a healthcare professional **first** ask you if you wanted to have invasive diagnostic testing?

1. In the same conversation / appointment that you received your screening results
2. In a subsequent conversation / appointment
3. Not sure
4. Thinking about the way that healthcare professionals supported you to consider whether or not to have invasive diagnostic testing for Down syndrome -

Would you say that:

|  | Yes | No | Not sure |
| --- | --- | --- | --- |
| - 1. . You understood that having invasive diagnostic testing is optional? |  |  |  |
| - 1. You got the impression that invasive diagnostic testing was a routine part of antenatal care? |  |  |  |
| - 1. You had enough opportunity to discuss with healthcare professional(s) whether or not to have invasive diagnostic testing? |  |  |  |
| - 1. You had enough time to decide whether or not to have invasive diagnostic testing? |  |  |  |
| - 1. You felt pressured by healthcare professionals to have invasive diagnostic testing? |  |  |  |

1. After you received your [combined or quadruple test / NIPT] (*auto-adjust text based on previous responses*) result, how much time were you given to decide whether you wanted to have invasive diagnostic testing?

I was asked to make my decision…

- 1. Immediately
  2. Within 1 - 2 days
  3. Within 3 - 6 days
  4. Within 1 – 2 weeks
  5. Within 3 - 4 weeks
  6. Within more than 1 month
  7. Not sure

Did you have invasive diagnostic testing for Down syndrome?

Yes > *to question 39*

No >*to section 12*

1. *(Only displayed if responded ‘Yes’ to q.38*) Did you receive a positive prenatal diagnosis of Down syndrome for your baby?
   1. Yes >*to section 13*
   2. No >*to section 15*

# SECTION 12: Questions about declining prenatal diagnostic testing (*only displayed to respondents who were offered and did not have diagnostic testing)*

Did you tell healthcare professionals that you did not wish to have invasive diagnostic testing for Down syndrome?

Yes

No > *skip to question 42*

Not sure

*(Only displayed if responded ‘yes’ to question 40)* After you told healthcare professionals that you did not wish to have invasive diagnostic testing, were you offered this again?

Yes

No

Not sure

41b. (*Only displayed if responded ‘yes’ to question 41*) How many additional times were you offered invasive diagnostic testing after you told healthcare professionals that you did not wish to have this?

Once

2-3 times

4 or more times

Not sure

1. To what extent did you feel that your decision not to have invasive diagnostic testing was respected by healthcare professionals?

My decision was:

- 1. Completely respected
  2. Mostly respected
  3. Somewhat respected
  4. Slightly respected
  5. Not respected at all
  6. Not sure

1. Would you like to share anything else about the care you received in relation to your choice not to have invasive diagnostic testing? If so, please write it in this box.

# SECTION 13: Questions about receiving prenatal diagnosis *(only displayed to respondents who received prenatal diagnosis)*

**These questions are about the way that healthcare professionals gave you the prenatal diagnosis of Down syndrome– that is, the results of your amniocentesis or chorionic villus sampling, which confirmed that your baby has Down syndrome**.

1. How was the news that your baby has Down syndrome first shared with you?
   1. In person >*to question 45*
   2. By telephone *>to question 46*
   3. By post/email *>* *to question 46*
   4. Other - Please specify: *>* *to question 46*
2. (*Displayed only if ‘In person’ is selected*) Was your partner present when the news was shared with you?
   1. Yes
   2. No
   3. Not sure
   4. Not applicable
3. Which healthcare professional first gave you the news?
4. Midwife > *to question 46b*
5. GP
6. Obstetrician or Gynaecologist (Doctor who specializes in pregnancy/ women’s reproductive health)
7. Genetic counsellor
8. Other Doctor
9. Nurse (e.g., practice nurse at GP surgery)
10. Receptionist / secretary / administrator
11. Not sure / can’t remember
12. Other (please specify):

46b. (*Only displayed if ‘midwife’ selected in previous question*) Which kind of midwife gave you the news that your baby has Down syndrome?

- 1. Community midwife
  2. Hospital midwife
  3. Screening midwife
  4. Private / Independent midwife
  5. Other
  6. Not sure

1. Did you feel that the news was presented to you mainly as:
   1. Positive / good news
   2. Neither positive nor negative news
   3. Negative / bad news
   4. Not sure
2. Thinking about the information that healthcare professionals gave you when discussing the diagnosis -

Do you feel you received the right amount of information about the following topics:

|  | Received too little information about this | Received the right amount of information about this | Received too much information about this | Not sure |
| --- | --- | --- | --- | --- |
| 1. Medical or physical aspects of Down syndrome |  |  |  |  |
| 1. Difficulties that children / people with Down syndrome may have (other than medical issues) |  |  |  |  |
| 1. Abilities that children / people with Down syndrome may have |  |  |  |  |
| 1. What everyday life may be like for families raising a child with Down syndrome |  |  |  |  |
| 1. Support available for families raising a child with Down syndrome |  |  |  |  |

1. Thinking about the information that healthcare professionals gave you when discussing the diagnosis -

Would you say the information you were given about Down syndrome was:

|  | Yes | No | Not sure |
| --- | --- | --- | --- |
| - 1. Balanced? - By this we mean presenting positive and negatives fairly and equally |  |  |  |
| - 1. Relevant to you? |  |  |  |
| - 1. Accurate? |  |  |  |
| - 1. Up to date? |  |  |  |
| - 1. Easy to understand? |  |  |  |

1. When you were given the diagnosis, did you receive information or support from any of the following organisations:

Select all that apply

- 1. Down’s Syndrome Association (DSA)
  2. Down’s Syndrome Scotland (DSS)
  3. Positive About Down Syndrome (PADS)
  4. Antenatal Results and Choices (ARC)
  5. Local support group
  6. Other – please specify: _____________________
  7. None of these
  8. Not sure

(*Possible to select any combination of* ***a, b, c, d****,* ***e,*** *and* ***f****)*

50b. (*Only displayed if respondents have selected at least one of the options a – f in question 50*) How helpful did you find the information or support you received from each organisation?

(*Only organisations selected in previous question are displayed)*

|  | Very helpful | Somewhat helpful | Not helpful | Not sure |
| --- | --- | --- | --- | --- |
| 1. Down Syndrome Association (DSA) |  |  |  |  |
| 1. Down’s Syndrome Scotland (DSS) |  |  |  |  |
| 1. Positive About Down Syndrome (PADS |  |  |  |  |
| 1. Antenatal Results and Choices (ARC) |  |  |  |  |
| 1. Local support group ____________ |  |  |  |  |
| 1. (*Other:)* |  |  |  |  |

1. Would you like to share anything else about your experiences of being given the prenatal diagnosis of Down syndrome? If so, please write it in this box

# SECTION 14: Questions about discussion of termination *(only displayed to respondents who received prenatal diagnosis)*

**These questions are about your experiences of healthcare professionals discussing the possibility of pregnancy termination with you.**

1. Did healthcare professionals mention the possibility of terminating your pregnancy:

Select all that apply

- 1. Before you received the diagnosis of Down syndrome
  2. During the same conversation / appointment that you received the diagnosis
  3. During a subsequent appointment / correspondence
  4. This was mentioned, but not sure when
  5. This was never mentioned

(*Possible to select* ***a****,* ***b****, and* ***c*** *together*)

52b. (*Displayed unless ‘never is selected in previous question*) At which points in your pregnancy was the possibility of having a termination mentioned to you?

Select all that apply

- 1. First trimester
  2. Second trimester
  3. Third trimester
  4. Not sure

(Possible to select **a, b,** and **c** together)

1. (*Display unless ‘Never’ is selected in previous question)* Did healthcare professionals directly ask you if you wanted a termination:

If you were asked more than once, please tell us about the first time you were asked.

- 1. During the same conversation /appointment that you received the diagnosis
  2. During a subsequent conversation / correspondence
  3. This was asked, but not sure when
  4. Never

Did you tell healthcare professionals that you did not wish to have a termination?

Yes

No

Not sure

1. (*Displayed unless answered ‘Never’ to question 52 or ’No’ to question 54*) After you told a healthcare professional that you were continuing your pregnancy, was the possibility of having a termination mentioned again during your maternity care?
   1. Yes
   2. No
   3. Not sure

60b. (*Displayed only if ‘Yes’ is selected in question 55*) How many additional times was the possibility of termination mentioned after you told healthcare professionals that you were continuing your pregnancy?

- 1. Once
  2. 2 or 3 times
  3. 4 or more times
  4. Not sure

Did you feel pressured by healthcare professionals to make a particular decision?

- 1. I felt pressured by healthcare professionals to terminate my pregnancy
  2. I felt pressured by healthcare professionals to continue my pregnancy
  3. I did not feel pressured by healthcare professionals to make a particular decision
  4. Not sure

1. To what extent do you feel that your decision to continue your pregnancy was respected by healthcare professionals?

My decision was…

1. Completely respected
2. Mostly respected
3. Neither respected nor disrespected
4. Mostly disrespected
5. Completely disrespected
6. Not sure
7. Would you like to share anything else about the way healthcare professionals supported you in your decision to continue your pregnancy after receiving the prenatal diagnosis? If so, please write it in this box.

# SECTION 15: Questions about receiving postnatal news (*only displayed to respondents who did not receive prenatal diagnosis*)

**These questions are about the time, after your baby’s birth, when healthcare professionals first told you that they thought your baby may have Down syndrome (not when it was confirmed by genetic testing).**

1. After your baby’s birth, when did healthcare professionals **first** discuss with you that he or she may have Down syndrome?
   1. Within 2 hours of birth
   2. 3 - 6 hours after birth
   3. 7 – 12 hours after birth
   4. 13 – 23 hours after birth
   5. 1 – 2 days after birth
   6. 3 – 6 days after birth
   7. 1 – 2 weeks after birth
   8. 3 – 4 weeks after birth
   9. Over 4 weeks after birth
2. How was the news that your baby may have Down syndrome first shared with you?
3. In person
4. By telephone
5. By post/email
6. Other - please specify:
7. (*Only displayed if ‘In person’ is selected in previous question*) Was your partner present when the news that your baby may have Down syndrome was first shared with you?
8. Yes
9. No
10. Not applicable
11. Not sure
12. Which healthcare professional **first** gave you the news that your baby may have Down syndrome?
13. Midwife *> to question 62b*
14. Obstetrician or Gynaecologist (Doctor who specializes in pregnancy/ women’s reproductive health)
15. Neonatologist or paediatrician (Doctor who specializes in care of babies / children)
16. GP
17. Other Doctor
18. Genetic counsellor
19. Nurse
20. I first raised this with healthcare professionals myself
21. Not sure
22. Other (please specify):

62b. (*Only displayed if ‘midwife’ is selected in previous question)* Which kind of midwife first gave you the news that your baby may have Down syndrome?

a. Community midwife

b. Hospital midwife

c. Screening midwife

d. Private / Independent midwife

e. Other

f. Not sure

1. Did you feel that the news was presented to you mainly as:
2. Positive / good news
3. Neither positive nor negative news
4. Negative / bad news
5. Not sure
6. Thinking about the information that healthcare professionals gave you when discussing the news that your baby may have Down syndrome –

Do you feel you received the right amount of information about the following topics:

|  | Received too little information about this | Received the right amount of information about this | Received too much information about this | Not sure |
| --- | --- | --- | --- | --- |
| 1. Medical or physical aspects of Down syndrome |  |  |  |  |
| 1. Difficulties that children / people with Down syndrome may have (other than medical issues) |  |  |  |  |
| 1. Abilities that children / people with Down syndrome may have |  |  |  |  |
| 1. What everyday life may be like for families raising a child with Down Syndrome |  |  |  |  |
| 1. Support available for families raising a child with Down Syndrome |  |  |  |  |

1. Thinking about the information that healthcare professionals gave you when discussing the news that your baby may have Down syndrome –

Would you say the information you were given about Down syndrome was:

|  | Yes | No | Not sure |
| --- | --- | --- | --- |
| 1. Balanced?  - By this we mean presenting positive and negatives fairly and equally |  |  |  |
| 1. Relevant to you? |  |  |  |
| 1. Accurate? |  |  |  |
| 1. Up to date? |  |  |  |
| 1. Easy to understand? |  |  |  |

1. When you were given the news after birth that your baby may have Down syndrome, did you receive information or support from any of the following organisations:

Select all that apply

- 1. Down’s Syndrome Association (DSA)
  2. Down’s Syndrome Scotland (DSS)
  3. Positive About Down Syndrome (PADS)
  4. Local support group
  5. Other – please specify: _____________________
  6. None of these
  7. Not sure

(*Possible to select any combination of* ***a, b, c,*** ***d*** *and* ***e****)*

66b. (*Only displayed if respondents have selected at least one of the options a - e in question 66*) How helpful did you find the information or support you received from each organisation?

(*Only organisations selected in previous question are displayed)*

|  | Very helpful | Somewhat helpful | Not helpful | Not sure |
| --- | --- | --- | --- | --- |
| 1. Down’s Syndrome Association (DSA) |  |  |  |  |
| 1. Down’s Syndrome Scotland (DSS) |  |  |  |  |
| 1. Positive About Down Syndrome (PADS) |  |  |  |  |
| 1. Local support group |  |  |  |  |
| 1. (*Other:)* ____________ |  |  |  |  |

- - - 1. Would you like to share anything else about your experiences of being given the news after birth that your baby may have Down syndrome? If so, please write it in this box.

# SECTION 16: Questions about overall maternity experiences (*All respondents)*

**These questions are about your overall experiences of maternity care in relation to your baby having Down syndrome.**

1. Would you say that:

|  | Yes | No | Not sure | Not applicable |
| --- | --- | --- | --- | --- |
| - 1. The **language used** by healthcare professionals when discussing your baby having (or possibly having) Down syndrome was respectful? |  |  |  |  |
| - 1. The **attitude shown** by healthcare professionals towards your baby having (or possibly having) Down syndrome was respectful? |  |  |  |  |
| - 1. The attitude shown by healthcare professionals towards your baby having (or possibly having) Down syndrome had a negative impact on your mental wellbeing? |  |  |  |  |
| - 1. You were given enough reassurance about the future for you, your baby and your family? |  |  |  |  |
| - 1. There was enough support available for your own mental wellbeing during your pregnancy? |  |  |  |  |
| - 1. You received enough information and support with planning for your baby’s delivery? |  |  |  |  |
| - 1. You received enough information and support around your baby’s care after birth? |  |  |  |  |
| - 1. Healthcare professionals were competent and confident in caring for your baby in regard to him or her having Down syndrome? |  |  |  |  |

1. Would you like to share anything that you feel could have improved your overall maternity experience in relation to your baby having Down syndrome? If so, please write it in this box.
2. Would you like to share anything that particularly helped you when you discovered your baby has or may have Down syndrome, and that you would recommend to others? If so, please write it in this box.

# SECTION 17: Sociodemographic questions (*all respondents*)

**These final questions are about your background and family situation. You do not have to answer them, but knowing this information will help us to consider whether people from different backgrounds are having different experiences of maternity care.**

1. What is your current marital status?

| Married/civil partnership and living with spouse/civil partner |
| --- |
| Living with partner |
| Divorced /Separated /Single /Widowed /Not currently living with partner |

70b. What was your marital status while you were pregnant with your child with Down syndrome?

| Married/civil partnership and living with spouse/civil partner |
| --- |
| Living with partner |
| Divorced /Separated /Single /Widowed /Not currently living with partner |

1. What sex is your child with Down syndrome?
   1. Male
   2. Female
2. **Before** having your child with Down syndrome, had you previously given birth?
   1. No
   2. Yes

73b. (*Display if ‘yes’ is selected in question 73)* How many children had you given birth to **before** having your child with Down syndrome?

I had previously given birth to Choose an item.

1. What is your ethnic group?

White

- 1. English / Welsh / Scottish / Northern Irish / British
  2. Irish
  3. Gypsy or Irish Traveller
  4. Any other White background

Mixed / Multiple ethnic groups

- 1. White and Black Caribbean
  2. White and Black African
  3. White and Asian
  4. Any other Mixed / Multiple ethnic background

 Asian / Asian British

- 1. Indian
  2. Pakistani
  3. Bangladeshi
  4. Chinese
  5. Any other Asian background

 Black / African / Caribbean / Black British

- 1. African
  2. Caribbean
  3. Any other Black / African / Caribbean background

 Other ethnic group

- 1. Arab
  2. Any other ethnic group

1. Do you regard yourself as belonging to any particular religion? If yes – which one?
   1. No religion
   2. Christian *> to question 75b*
   3. Buddhist
   4. Hindu
   5. Jewish
   6. Muslim
   7. Sikh
   8. Any other religion

75b. (*Display only* *if ‘Christian’ is selected in previous question*) Which Christian denomination do you belong to?

1. Roman Catholic
2. Anglican / Church of England / Church of Scotland / Church in Wales
3. Presbyterian
4. Methodist
5. Baptist
6. Christian – no denomination
7. Other Christian denomination
8. What is the highest educational qualification you have obtained?
   1. Postgraduate university degree (MA, MSc, PhD, etc.)
   2. Undergraduate university degree (BA, BSc, etc.)
   3. Upper secondary school qualification (A-level or equivalent)
   4. Secondary school qualification (GCSE or equivalent)
   5. None of these
9. How well would you say you [ ‘and your partner’ *auto-insert* *if applicable*] are managing financially these days?

Would you say you are...

| Living comfortably |  |
| --- | --- |
| Doing alright |  |
| Just about getting by |  |
| Finding it quite difficult |  |
| Finding it very difficult |  |

1. Suppose you only had one week to raise £2000 for an emergency, which of the following best describes how hard it would be for you to get that money?

| I could easily raise the money |  |
| --- | --- |
| I could raise the money, but it would involve some sacrifices (e.g. reduced spending, selling a possession) |  |
| I would have to do something drastic to raise the money (e.g. selling an important possession) |  |
| I don’t think I could raise the money |  |

# END OF SURVEY message (*all respondents)*

Thank you for your time spent taking this survey. Your responses have been recorded and are very much appreciated.

If you have any questions or comments about this research, please contact [removed for blind review].”

**Support organisations**

This survey includes questions about experiences which parents may have found emotional and unexpected. The following are organisations you may wish to contact for support.

**Support for parents of children with Down syndrome**:

Positive About Down Syndrome – Supporting and connecting parents and expectant parents of children with Down syndrome. <https://positiveaboutdownsyndrome.co.uk/>

Down’s Syndrome Association – Support for people with Down syndrome, their families and carers. <https://www.downs-syndrome.org.uk/> - 0333 1212300

**Support for parents of children with disabilities:**

Contact – Support for families with disabled children. <https://contact.org.uk/> - 0808 808 3555

Mencap –Support for people with learning disabilities and their families. <https://www.mencap.org.uk/> - 0808 808 1111

Kids – Support and services for children and young people with disabilities and their families. <https://www.kids.org.uk/>

**Other support**

For information about accessing statutory educational or social services to support your child and family, contact your local authority. Visit <https://www.gov.uk/find-local-council> to find their website.

If you feel you need help with your mental wellbeing, speak to your GP who will be able to direct or refer you to appropriate support.
